# Validation of the German version of the Protein Screener 55+

**DOI:** 10.1038/s41430-023-01266-y

**Published:** 2023-01-26

**Authors:** Eva Kiesswetter, Hanna M. Siebentritt, Daniel Schoene, Robert Kob, Ellen Freiberger, Cornel C. Sieber, Marjolein Visser, Hanneke A. H. Wijnhoven, Dorothee Volkert

**Affiliations:** 1 Institute for Biomedicine of Aging, Friedrich-Alexander-Universität Erlangen-Nürnberg, Nuremberg, Germany; 2 Institute of Medical Physics, Friedrich-Alexander-Universität Erlangen-Nürnberg, Erlangen, Germany; 3 Institute of Exercise and Public Health, Leipzig University, Leipzig, Germany; 4 Department of Medicine, Kantonsspital Winterthur, Winterthur, Switzerland; 5 Department of Health Sciences, Faculty of Science, and Amsterdam Public Health Research Institute, Vrije Universiteit Amsterdam, Amsterdam, The Netherlands

**Keywords:** Risk factors, Geriatrics

## Abstract

**Background/Objectives:**

The Protein Screener 55 + (Pro55 + ) is a brief food questionnaire to screen older community-dwelling adults for low protein intake. The result is the predicted probability of protein intake <1.0 g/kg adjusted body weight (aBW)/d ranging from 0–1. For purposes of cross-cultural validation, we translated the Pro55+ into German and tested its discriminative accuracy in detecting low protein intake of older community-dwelling people in Germany.

**Subjects/Methods:**

After translation and pilot-testing, the Pro55+ and the reference standard (3-day dietary record) were completed by 144 participants (81.6 ± 3.9 years, 61.8% female). Discriminative properties were tested by receiver operating characteristic curves and by calculating sensitivity and specificity for different cut-offs of predicted probability (>0.3/>0.5/>0.7) using <1.0 or <0.8 g/kg aBW/d to define low protein intake.

**Results:**

Protein intake was <1.0 g/kg aBW/d in 39.6% of the sample and <0.8 g/kg aBW/d in 17.4%. Area under the curve was 62.0% (95%CI 52.6–71.5) and 68.8% (58.1–79.4), respectively. Specificity was 82–90% using probability cut-offs of 0.5 and 0.7 for both protein thresholds. Sensitivity was poor for protein threshold of 1.0 g/kg aBW/d regardless of the used probability cut-offs. For protein threshold of <0.8 g/kg aBW/d, sensitivity was 88.0% (71.8–96.9) using a probability cut-off of 0.09.

**Conclusion:**

The overall discriminative accuracy of the German Pro55+ to identify older community-dwelling people with low protein intake was poor. However, applying different probability cut-offs allows increasing specificity and sensitivity for 0.8 g/kg aBW/d to levels justifying the use for certain purposes e.g. excluding individuals with adequate protein intake. Further validation is needed.

## Introduction

Low protein intake has been shown to be associated with accelerated loss of muscle mass and function in older people [1, 2]. Moreover, because of inflammatory processes, insulin resistance, reduced postprandial availability of amino acids, and a blunted anabolic response towards ingested protein, older people may have higher dietary protein needs compared to younger people [3, 4]. Therefore, in recent years expert groups have questioned the existing recommendation for protein intake of 0.8 g/kg BW/d and suggested an optimal protein intake of 1.0–1.2 g/kg BW/d for healthy older adults [5, 6]. In 2017, the German Nutrition Society changed their reference value on protein intake for healthy people 65 years or older to an estimated value of 1.0 g/kg BW/day [7].

Protein intake of older people is often below 1.0 g/kg BW/day [8, 9]. In a recent meta-analysis of four large cohort studies (*n* = 8107) in community-dwelling older people aged 55 years and older, the pooled prevalence of protein intake below 1 g/kg adjusted body weight (aBW)/d was 46.7% (95%CI 38.3–55.3) [10].

However, intervention studies using protein supplements have often failed to improve muscle mass and function in older people, presumably caused by sufficiently high habitual protein intake [11, 12]. Therefore, it is important to identify older people with low protein intake who may benefit from increased intake.

The adequacy of protein intake is usually evaluated by repeated 24 h recalls, dietary records or food frequency questionnaires (FFQ) [13]. The application of these methods and data analysis requires specific nutritional knowledge and therefore, needs to be performed by trained staff like dietitians or nutritionists. Furthermore, these methods are time-consuming and depend on the motivation and cooperation of the older individual. Especially in frail older adults, dietary assessment can be challenging due to physical limitations and recall bias caused by memory impairments [13–15]. Therefore, for clinical practice validated screening instruments for low protein intake would be helpful as a first step to reduce the number of the time-consuming full assessments.

In 2018, the Protein Screener 55 +, a brief food questionnaire to screen older community-dwelling adults (≥55y) for low protein intake (<1.0 g/kg aBW/d) with good discriminative ability compared to the reference standard a FFQ was developed by a Dutch research group [16]. As protein intake pattern may vary between countries, cross-cultural validation is needed before applying this tool in clinical practice in other countries.

The current study aimed to validate the German version of the Protein Screener 55+ in community-dwelling older people using 3-day-dietary-records as the reference standard.

## Material/subjects and methods

### Protein Screener 55+

The Protein Screener 55 + (https://proteinscreener.nl/#/) comprises ten questions on the amount of intake of protein-rich foods on an average day or the frequency of intake referring to the last four weeks. It includes additionally information on age, sex, body height and weight [16]. The items were derived from a semi-quantitative FFQ (HELIUS FFQ [17]) and selected using multivariable backward regression analyses [16]. The questions ask for slices of bread (number), glasses of milk, buttermilk or soymilk (number), meat with warm dish (portion size), cheese (amount and frequency), dairy products (frequency), eggs, (frequency), pasta (frequency), fish (frequency) and nuts/peanuts (frequency). The screening result is the predicted probability of a low protein intake (<1.0 g/kg aBW/d) with a higher score indicating a higher probability. A predicted probability of more than 0.3 has been identified as the optimal cut-off in a Dutch population, balancing sensitivity and specificity best, to screen for protein intake <1.0 g/kg aBW/d (sensitivity: 82.2%, specificity: 80.0%, AUC: 85.6%) [16].

### Validation process

We conducted the validation of the German version of the Protein Screener 55+ in a two-step approach.

#### Step 1: Translation and layout

The English version was translated to German by a researcher fluent in both languages (EK). Afterwards, two experts reviewed the German translation (HS, DV) for accuracy and comprehensibility. The consented version was then back translated to English by another researcher fluent in both languages (DS), who was blinded to the original version. All results were compared to the original version of the Protein Screener and consensus on the German version was reached.

As we aimed to use the Protein Screener as paper and pencil method that can be self-completed by older adults, a questionnaire template was designed focusing on readability and clear layout. In a pretest, four older adults (3 female, 1 male) aged between 70 and 86 years filled in the Protein Screener and completed a feedback questionnaire on comprehensibility, clarity, missing information, readability, format, time needed for completion, and further remarks. Based on this feedback, the content and layout of the questionnaire were slightly adapted including a simplification of the wording of question 10 and the presentation of answer categories of questions 1, 2 and 10. The time for completion (~5 min) was considered adequate. The German version of the Protein Screener 55+ can be found at https://proteinscreener.nl/#/.

#### Step 2: Validation study

The validation was conducted at the German study center (Institute for Biomedicine of Aging in Nuremberg) of two at the time ongoing studies (SPRINT-T [18] and SCOPE [19]) between July 2019 and February 2020. SPRINT-T is a multicenter randomized controlled trial to test the efficacy of a multicomponent intervention to prevent mobility disability in older adults (≥70 years) with physical frailty and sarcopenia [18]. For the current analysis, data from the final study visit were used. The SCOPE study is a multicenter prospective cohort study in community-dwelling adults (≥75 years) [19]. Data for the present analysis refer to the 24-month visit.

For the present analysis, only participants without major cognitive impairment as defined by a Mini Mental Status Examination (MMSE) score of ≥24 points of max. 30 points, plausible energy intake (mean energy intake ±3 standard deviations of sample mean) and with complete data on both the Protein Screener and the reference method (dietary record), were included. For the purpose of this validation study no a priori sample size calculation was performed.

As reference method, an estimated consecutive 3-day-dietary-record was used [8]. The dietary records did not include predefined food groups but had open fields to list all foods eaten. Participants were advised to stick to their usual eating habits and to report all consumed foods and beverages as detailed as possible, including portion sizes (e.g. grams, usual household measures, packaging information), fat content (e.g. milk, yogurt), ingredients and quantities of recipes, cooking methods and timing of consumption.

At the study visits, participants received both the Protein Screener and the dietary record with detailed oral and written instructions for completion at home during the week after the visit. For the dietary record, the three days for completion were determined a priori together with the participants. The completed documents were sent back in prepaid envelopes and checked for completeness and plausibility by a nutrition scientist or dietitian. Missing or implausible information was clarified by phone within one week after arrival. Data of the 3-day dietary records were entered in EBISpro-software (EBISpro, Willstätt-Legelshurst, Germany, 2016) by a trained nutrition scientist or dietitian to calculate intake of protein (g), and in addition of energy (kilocalories (kcal)), fat (g) and carbohydrates (g) per day based on the German nutrient database “Bundeslebensmittelschlüssel” (version 3.02, Karlsruhe, Germany). All entered data were cross-checked by a second nutrition scientist and mean values of the three days were used for analysis.

Protein intake is presented in g/kg aBW/d to consider reduced protein needs per kg body weight in overweight and increased protein needs per kg body weight in underweight persons [20]. Adjusted body weight was calculated for persons with BMI > 27 kg/m² and <22 kg/m² by using the body weight that corresponds to a BMI of 27 kg/m² or 22 kg/m², respectively [16]. Low protein intake was defined by either <1.0 g/kg aBW/d or <0.8 g/kg aBW/d – the same thresholds as described by Wijnhoven et al. (2018).

### Further measurements

#### Participants’ characteristics

Sex, age, and living situation (living alone vs. living with others) were assessed through standardized questionnaires. Illness burden was evaluated by the study physician using the Cumulative Illness Rating Scale for Geriatrics (CIRS-G), rating 14 disease categories on a scale from 0 (no problem) to 4 (extremely severe) [21].

Functional status was measured using the Short Physical Performance Battery (SPPB; 0–12 points) with tests on standing balance, usual gait speed, and lower extremity strength (chair rise test) [22]. An SPPB score ≤9 points was defined as poor performance [23].

Handgrip strength (kg) was measured with a JAMAR hydraulic hand dynamometer (Sammons Preston Rolyan, Bolingbrook, IL, USA) in a seated position according to the study specific standard operating procedures. The maximal value of the dominant hand was used.

For calculating body mass index (BMI, kg/m²) body weight was measured in light clothes with calibrated scales and body height with a stadiometer. Nutritional status was evaluated using the Mini Nutritional Assessment Short Form (MNA-SF, 0–14 points) and categorized as normal (12–14 points), at risk of malnutrition (8–11 points), and malnourished (0–7 points) [24].

### Statistical analyses

Participant characteristics and intake data are presented as relative frequencies for nominal and ordinal variables or as mean±standard deviation for continuous variables for the total sample and stratified by protein intake (<, ≥1.0 g/kg aBW/d) and gender. Group differences were tested by the Mann–Whitney-U-Test or Chi²-test. To identify potential country differences, the questionnaire responses were compared to the original validation study from the Netherlands using the provided data of the Longitudinal Aging Study Amsterdam (LASA) (*n* = 1348).

For the validation of the German version of the Protein Screener 55+, we calculated the predicted probability of low protein intake for each participant by using the equation provided by Wijnhoven et al. (2018) (supplementary material 1). We tested the performance of the equation using receiver operating characteristic (ROC) curves. Discriminative ability is considered good with an area under the curve (AUC) > 0.8. Sensitivity, specificity, positive predictive value (PPV) and negative predictive value (NPV) were calculated for different probability cut-offs (0.3, 0.5, 0.7) and using <1 g protein/kg aBW as reference value. An additional analysis was conducted with <0.8 g protein/kg aBW to define low protein intake.

Statistical analyses were performed with SPSS version 26 (IBM SPSS Statistics, Chicago, IL, USA).

## Results

### Participants

The Protein Screener was completed by 161 participants. Seventeen participants were excluded from analysis due to MMSE score <24 points (*n* = 4), tube feeding (*n* = 1), implausible energy intake (*n* = 1) or incomplete data (*n* = 11). Therefore, the final sample comprised 144 participants.

Mean age of participants was 81.6 ± 3.9 years, nearly two thirds were female (61.8%) and one third (34%) had a poor functional status (Table 1). Participants’ characteristics did not differ between groups with low and sufficient protein intake (Table 1).

**Table 1:** Participants’ characteristics for total sample and stratified by actual protein intake based on the 3-day dietary record.

	Total (*n* = 144)	<1.0 g/kg aBW/d (*n* = 57)	≥1.0 g/kg aBW/d (*n* = 87)	*p*-value
Age [years]	81.6 ± 3.9	81.3 ± 3.7	81.7 ± 4.1	0.671
Female sex [%]	61.8	61.4	62.1	0.936
Living alone [%]	66.0	70.2	63.2	0.389
MMSE [points]	28.0 ± 1.5	28.1 ± 1.7	28.0 ± 1.4	0.506
SPPB [points]^a^	9.8 ± 2.3	10.0 ± 2.3	9.7 ± 2.4	0.420
SPPB ≤ 9 points [%]^a^	34.0	29.6	36.8	0.384
HG Strength [kg]^a^	25.8 ± 9.8	26.2 ± 8.9	25.5 ± 10.4	0.371
CIRS [categories]	4.2 ± 2.1	4.3 ± 2.1	4.0 ± 2.0	0.413
CIRS Total	7.5 ± 3.2	7.7 ± 3.5	7.4 ± 3.2	0.807
MNA-SF [%]				
Normal	82.6	84.2	81.6	0.472
At risk	15.3	12.3	17.2	
Malnourished	2.1	3.5	1.1	
BMI [kg/m²]	28.0 ± 5.1	28.3 ± 4.4	27.8 ± 5.5	0.241
BMI < 22 kg/m² [%]	8.3	3.5	11.5	0.125
BMI ≥ 30 kg/m² [%]	25.7	29.8	23.0	0.359

*HG* Handgrip, *CIRS* Cumulative Illness Rating Index, *MNA-SF* Mini Nutritional Assessment Short Form, *BMI* Body Mass Index. *aBW* adjusted body weight, *MMSE* Mini Mental State Examination (max. 30 points), *SPPB* Short Physical Performance Battery (max. 12 points).

^a^*n* = 141, Continuous variables: mean±standard deviation, Mann–Whitney-U-Test; categorical/nominal variables: Chi²-Test.

### Dietary intake

Mean energy intake was 1770.1 ± 440.4 kcal/d and was higher in those with sufficient protein intake than in those with low protein intake (Table 2) as well as in males than in females (supplementary material 2). Mean protein intake amounted 1.0 ± 0.3 g/kg aBW/d. Protein intake <1.0 g/kg aBW/d was present in 39.6% of the sample and <0.8 g/kg aBW/d in 17.4%, respectively.

**Table 2:** Intake of energy and macronutrients for total sample and stratified by actual protein intake based on the 3-day dietary record.

Dietary Intake	Total (*n* = 144)	<1.0 g/kg aBW/d (*n* = 57)	≥1.0 g/kg aBW/d (*n* = 87)	*p*-value
Energy [kcal/d]	1770.1 ± 440.4	1489.2 ± 356.6	1954.1 ± 391.4	<0.001
Carbohydrates [E%]	41.8 ± 7.2	42.6 ± 7.7	41.3 ± 6.7	0.174
Fat [E%]	38.8 ± 6.8	38.4 ± 6.5	38.9 ± 6.9	0.509
Protein [g/d]	67.7 ± 17.3	52.6 ± 11.8	77.5 ± 12.5	<0.001
Protein [E%]	15.9 ± 2.9	14.8 ± 2.8	16.5 ± 2.8	0.001
Protein [g/kg aBW/d]	1.0 ± 0.3	0.7 ± 0.2	1.2 ± 0.2	<0.001
Protein < 0.8 g/kg aBW/d [%]	17.4	43.9	0.0	<0.001

Continuous variables: mean±standard deviation, Mann–Whitney-U-Test; categorical/nominal variables: Chi²-Test; aBW adjusted body weight.

### Protein Screener

The result of the Protein Screener, expressed as median (interquartile range) predicted probability, was 0.17 (0.04–0.46). In participants with protein intake <1.0 g/kg aBW/d the predicted probability was significantly higher compared to those with sufficient protein intake (0.31 (0.08–0.68) vs. 0.13 (0.02–0.41), *p* = 0.015). The responses to the single items of the Protein Screener in comparison to the Dutch validation study are presented in supplementary material 3.

### Validation

**Fig. 1:**
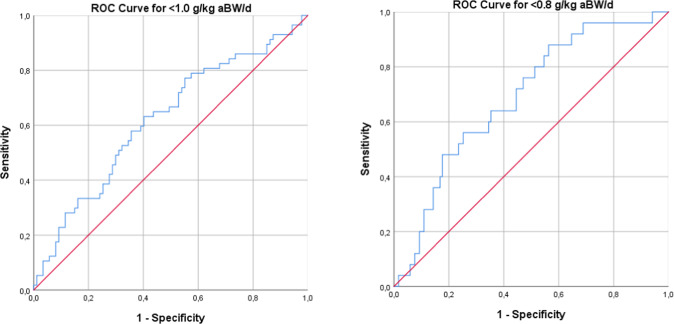
Receiver-operating characteristic (ROC) curves showing the discriminative ability of the German Version of Protein Screener 55 + (predicted probability of protein intake <1 g/kg adjusted body weight (aBW)/d). Left panel with <1 g protein/kg aBW/d as reference, right panel with <0.8 g protein/g aBW/d as reference.

When applying a protein intake of 1.0 g/kg aBW/d as reference value, the AUC was 62.0% (95%CI 52.6–71.5), indicating low discriminative abilities of the Protein Screener (Fig. 1). Highest sensitivity + specificity was found for the probability cut-off value of 0.17 (sensitivity: 63.2% (95%CI 50.3–74.9); specificity: 57.5% (95%CI 47.0–67.5); PPV 49.3% (95%CI 38.0–60.7); NPV 70.4% (95%CI 59.2–80.2)). Sensitivity, specificity, PPV and NPV for further probability cut-off values are presented in Table 3. Sensitivity of the Protein Screener to identify participants with protein intake <1.0 g/kg aBW/d was generally low. Moderate specificity was identified for probability cut-offs 0.5 (specificity: 83.9% (95%CI 75.3–90.6)) and 0.7 (89.7% (95%CI 82.1–94.9)).

**Table 3:** Sensitivity, Specificity, Positive Predictive Value (PPV) and Negative Predictive Value (NPV) with protein intake < 1.0 g/kg adjusted body weight (aBW)/d as the reference for 3 different cut-off probabilities (*n* = 144).

	Probability					
	≤0.3	>0.3	≤0.5	>0.5	≤0.7	>0.7
≥1.0 g protein/kg aBW/d	*n* = 58	*n* = 29	*n* = 73	*n* = 14	*n* = 78	*n* = 9
<1.0 g protein/kg aBW/d	*n* = 27	*n* = 30	*n* = 38	*n* = 19	*n* = 44	*n* = 13
Cut-off probability	**>0.3**		**>0.5**		**>0.7**	
Sensitivity	52.6 (39.8–65.3)		33.3 (22.0–46.1)		22.8 (13.3–34.7)	
Specificity	66.7 (56.4–76.0)		83.9 (75.3–90.6)		89.7 (82.1–94.9)	
PPV	50.8 (38.3–63.4)		57.6 (40.6–73.4)		59.1 (38.4–77.8)	
NPV	68.2 (57.9–77.5)		65.8 (56.7–74.2)		63.9 (55.2–72.1)	

Percentage (95% Confidence Interval).

When using 0.8 g/kg aBW/d as reference value, the AUC was 68.8% (95%CI 58.1–79.4)) (Fig. 1). Highest sensitivity + specificity was found for the probability cut-off of 0.09 (Sensitivity: 88.0% (95%CI 71.8-96.9); Specificity: 42.0% (95%CI 33.4–51.0); PPV 24.2% (95%CI 16.2–33.6); NPV 94.3% (95%CI 86.0–98.6)). Specificity could be increased to a moderate level, when using the probability cut-offs 0.5 and 0.7 (Table 4).

**Table 4:** Sensitivity, Specificity, Positive Predictive Value (PPV) and Negative Predictive Value (NPV) with protein intake < 0.8 g/kg adjusted body weight (aBW)/d as the reference for 3 different cut-off probabilities (*n* = 144).

	Probability					
	≤0.3	>0.3	≤0.5	>0.5	≤0.7	>0.7
≥0.8 g protein/kg aBW/d	*n* = 76	*n* = 43	*n* = 98	*n* = 21	*n* = 104	*n* = 15
<0.8 g protein/kg aBW/d	*n* = 9	*n* = 16	*n* = 13	*n* = 12	*n* = 18	*n* = 7
Cut-off probability	**>0.3**		**>0.5**		**>0.7**	
Sensitivity	64.0 (44.5–80.8)		48.0 (29.3–67.1)		28.0 (13.1–47.2)	
Specificity	63.9 (55.0–72.1)		82.4 (74.9–88.5)		87.4 (80.7–92.5)	
PPV	27.1 (16.9–39.3)		36.4 (21.4–53.3)		31.8 (15.1–52.5)	
NPV	89.4 (81.7–94.8)		88.3 (81.5–93.4)		85.2 (78.2–90.8)	

Percentage (95% Confidence Interval).

## Discussion

We have translated the Protein Screener 55+ to German and tested its discriminative accuracy in detecting low protein intake of older community-dwelling people living in the Region of Nuremberg in Southern Germany. Based on the AUCs, the German version’s ability to discriminate between low and sufficient protein intake – for both 1.0 g/kg aBW/d and 0.8 g/kg aBW/d thresholds – was poor. The 0.3-probability cut-off that was recommended based on the best trade-off between sensitivity and specificity in the original validation study [16], did not distinguish well between older people with low and sufficient protein intake in our study. We were not able to identify probability cut-off values with moderate or high levels of both sensitivity and specificity.

As the result of the Protein Screener is expressed as predicted probability, that can take values between 0 and 1, it is possible to use specific cut-off values according to the purpose of the screening (i.e. ruling in or ruling out a condition) [16, 25]. A low probability cut-off should be used when high sensitivity is important, meaning not to miss any individuals with low protein intake [16, 25]. In our study, with a very low probability cut-off of 0.09 (best trade-off between sensitivity and specificity), it was possible to classify 88% of the older people with protein intake below 0.8 g/kg aBW/d correctly. With regard to the reference threshold of 1.0 g/kg aBW/d, the sensitivity of the Protein Screener was poor – even when applying low probability cut-offs. The slightly better performance of the Protein Screener when using the lower protein threshold might be explained by the fact that it is easier to detect more severe stages of a condition (<0.8 g/kg aBW/d) [26]. The use of higher probability cut-offs increases specificity and is recommended when it is crucial to have few false positive cases [16, 25]. For purposes of identifying older individuals with sufficient protein intake, the Protein Screener seems somewhat better suited. When applying probability cut-offs of 0.5 or 0.7, specificity reached 82–90% in the current sample (Tables 3–4). From a public health perspective, the screener may thus be used to filter out individuals with sufficient protein intake. The remaining individuals would then need a detailed dietary assessment to determine the actual protein intake.

Compared to the Dutch validation study [16] the discriminative properties of the German version were distinctly lower. These deviating results are likely caused by several reasons that are discussed in the following.

Firstly, some methodological differences regarding the used reference method need to be acknowledged. Originally, the Protein Screener was developed data-driven based on the semi-quantitative HELIUS FFQ, which refers to the consumption of food items in the last four weeks [17]. Accordingly, the same items are incorporated in the Protein Screener as well as in the original reference standard, which may partially explain the higher agreement between these tools [26]. In the present study, a 3-day dietary record was used as reference standard that documents current intake. Albeit the Protein Screener and the dietary record were assessed in parallel, the periods the intake data refer to did not overlap, which may have affected the results. However, eating habits of older people are generally considered relatively stable [27, 28]. Moreover, the agreement between FFQs and dietary records as methods to assess protein intake was characterized as low to adequate in previous studies [29–34]. The HELIUS FFQ was specifically validated in older people (72 ± 9 years), and for intake of energy and macronutrients, an acceptable to good relative validity compared to 24 h recalls was described [35]. For protein intake, the group level bias between the methods was 4.7% and the Pearson correlation coefficient amounted to 0.39 [35]. On the level of food intake, the HELIUS FFQ overestimated the intake of fish, eggs, dairy products and nuts/seeds compared to the 24 h recalls items that are also included in the Protein Screener. It can be assumed, that 24 h recalls and 3-day dietary records may not depict usual intake of food groups such as fish and eggs that are typically not eaten daily [35].

Secondly, participant selection may have affected test accuracy [26]. The sample in the present study had a distinctly higher mean age compared to that of the Dutch validation study (82 ± 4 years vs. 62 ± 4 years) [16]. Even though cognitive status was overall good – expressed by a mean MMSE score of 28 points – completing the questionnaires might be more challenging for people of advanced age bearing the risk of a recall bias [36]. A format, where the screener is conducted as an interview by trained personnel might be an option to increase accuracy. The higher age might also have affected portion sizes and eating behavior due to decreased appetite, earlier satiety or functional impairments [27, 37–39]. Therefore, the portion sizes taken as basis for the screener might be larger compared to real portions of our participants. Further, the proportion of older people with low protein intake was higher in the present study compared to the Dutch validation study (<1.0 g/kg aBW/d: 40% vs. ~30%; <0.8 g/kg aBW/d: 17% vs. ~10%), which may also be explained by aspects like higher age and limited functional abilities [40, 41]. The high prevalence of older people with low protein intake as observed in our study underlines the need for appropriate screening tools. One quarter of participants in our study was obese; the proportion was slightly higher in those with low protein intake while energy intake was lower in participants with obesity. Even though we checked all dietary records thoroughly, this could be an indicator for underreporting in older people with obesity, which has been described previously [42]. However, sensitivity and specificity in subgroups of participants with BMI < and ≥30 kg/m² did not differ markedly (data not shown).

Thirdly, potential cultural differences in food intake between older people from Germany and the Netherlands may have affected the results. Comparing the responses to the single Protein Screener items between the Dutch and the current study (supplementary material 3) indicates that the chosen protein sources are relevant for protein intake in both countries. Some differences e.g. in the portion size of meat with a warm dish might be explained by different sample compositions regarding sex and age. Two recent publications from Germany (D) and the Netherlands (NL) showed similar contributions of animal and plant protein to total daily protein intake in older adults with a mean age >70 years [43, 44]. In both studies, main protein sources were meat/meat products (NL: 23% vs. D: 24%), cereals/starchy foods (NL: 19% vs. D: 21%) and dairy products (NL: 26% vs. D: 20%) [43, 44]. The contribution of dairy products appeared to be slightly higher in Dutch compared to German older people. A recent analysis of the SHARE database supports this assumption reporting a higher frequency of dairy product consumption at every day of the week in Dutch compared to German older adults [45]. However, this difference could not be seen in the answers to the four Protein Screener items referring to dairy products. For few items e.g. pasta, the category referring to the most frequent consumption was more pronounced in our compared to the Dutch study, which might have led to an underestimation of protein intake by this source. However, this effect is considered marginal. It remains to be investigated if exchanging certain items of the Protein Screener by protein sources more commonly consumed in Germany e.g. potatoes, legumes or cold meat could improve the precision of the German Screener version.

Further, as no a priori sample size calculation was performed, a too small sample size might have influenced the validation results. However, following the guidance for determining the minimum sample size required for a screening study [46], a minimum sample size of 122 participants, including *n* = 49 with low protein intake as determined by our reference standard (3-day dietary record; prevalence of ~40% for low protein intake <1 g/kg aBW/d), would have been sufficient to achieve 80% power for detecting a change in the percentage value of sensitivity from 50% (chance) to 70% based on an alpha error level of 5%.

## Conclusions

The overall discriminative accuracy of the German version of Protein Screener 55+ to identify older community-dwelling people with low protein intake was poor compared to the reference standard of a 3-day dietary record. However, applying different probability cut-offs increased specificity and sensitivity for 0.8 g/kg aBW/d to sufficient levels justifying the use for certain purposes e.g. identifying individuals with sufficient intake in order to exclude them from an in-depth and time-consuming nutritional assessment to determine actual protein intake. Further validation studies in samples with a broader age range, using different reference methods, applying modified versions by exchanging single items or comparing different application methods i.e. paper-pencil vs. interview are needed.

## Supplementary information

Supplementary Materials 1-3

## Data Availability

Data described in the manuscript are available from the corresponding author on reasonable request.
