# Supplementary material for: Validation of the German version of the Protein Screener 55+
Source: Eur J Clin Nutr. 2023 Jan 26;77(5):579–85. doi: 10.1038/s41430-023-01266-y (PMC10169657; doi:10.1038/s41430-023-01266-y)
Supplement: Supplementary file 1 — Supplementary Materials 1-3 [file 41430_2023_1266_MOESM1_ESM.pdf]

Supplementary materials

Validation of the German version of the Protein Screener 55+

Eva Kiesswetter, Hanna M. Siebentritt, Daniel Schoene, Robert Kob, Ellen Freiberger,

Cornel C. Sieber, Marjolein Visser, Hanneke A.H. Wijnhoven, Dorothee Volkert

European Journal of Clinical Nutrition

Supplementary material 1 describes the steps to calculate the predicted probability of low protein intake according to the Protein Screener 55+. Supplementary material 2 presents the dietary intake data of the sample of older community-dwelling adults stratified by gender. Supplementary material 3 shows the responses to the single items of the Protein Screener of the German sample in comparison to the Dutch validation study.

Supplementary Material 1: SPSS Syntax for the equation to calculate the predicted probability of low protein intake (Protein Screener 55+)

1. **Step: Calculate adjusted body weight (weight_adj) according to Berner et al 2013:**

<u>Variables needed:</u>

Body Mass Index: BMI (kg/m²)

Age: Age (years)

Body height: height_m (meter)

Body weight: weight (kg)

<u>Equations:</u>

```
IF  (BMI < 18.5 & Age < 71) weight_adj=(height_m) * (height_m) * 18.5.

EXECUTE.

IF  (BMI > 25.0 & Age < 71) weight_adj=(height_m) * (height_m) * 25.

EXECUTE.

IF  (BMI >= 18.5 & BMI <= 25 & Age < 71) weight_adj=weight.

EXECUTE.

IF  (BMI < 22.0 & Age >= 71) weight_adj=(height_m) * (height_m) * 22.

EXECUTE.

IF  (BMI > 27.0 & Age >= 71) weight_adj=(height_m) * (height_m) * 27.

EXECUTE.

IF  (BMI >= 22.0 & BMI <= 27.0 & Age >= 71) weight_adj=weight.

EXECUTE.
```

2. **Step: Recode the original Protein Screener Questions**

Variables needed:

Protein Screener Questions (The "d" at the end of the variable names indicates "per day")

Slices of bread: amount_slice_breadd (1 = less than 1 slice *until* 14 = >12 slices)

Glasses of milk, buttermilk or soymilk: amount_milkd (1 = less than 1 glass *until* 14 = >12 glasses)

Meat with warm dish: amount_meatd (0 = not applicable, does not eat meat *until* 5 = 3/4 plate)

Frequency dairy products: dairy_dessert (1 = not in these 4 weeks *until* 10 = 7 days/week)

Frequency eggs: freq_eggd (1 = not in these 4 weeks *until* 10 = 7 days/week)

Frequency pasta: freq_pastad (1 = not in these 4 weeks *until* 10 = 7 days/week)

Frequency fish: freq_fishd (1 = not in these 4 weeks *until* 10 = 7 days/week)

Frequency nuts/peanuts: freq_peanutsd (1 = not in these 4 weeks *until* 10 = 7 days/week)

Frequency cheese on bread: cheese_on_bread (1 = not in these 4 weeks *until* 10 = 7 days/week)

Slices of bread with Cheese: bread_with_cheese (1 = less than 1 slice *until* 14 = >12 slices)

** Recode slices of bread (amount_slice_breadd).*

RECODE amount_slice_breadd (1 thru 3 = 1) (4=2) (5=1) (6 thru highest=1)
    (MISSING=SYSMIS) INTO amount_slice_breadd1.

EXECUTE.

RECODE amount_slice_breadd (1 thru 3 = 1) (4=1) (5=2) (6 thru highest=1)
    (MISSING=SYSMIS) INTO amount_slice_breadd2.

EXECUTE.

RECODE amount_slice_breadd (1 thru 3 = 1) (4=1) (5=1) (6 thru highest=2) (MISSING=SYSMIS) INTO amount_slice_breadd3.

EXECUTE.

** Recode glasses of milk (amount_milkd).*

RECODE amount_milkd (1=1) (2=2) (3 thru highest=1) (ELSE=SYSMIS) INTO amount_milkd1.

EXECUTE.

RECODE amount_milkd (1=1) (2=1) (3 thru highest=2) (ELSE=SYSMIS) INTO amount_milkd2.

EXECUTE.

**Recode amount meat warm meal (amount_meatd).*

RECODE amount_meatd (0=1) (1=1) (2=2) (3=1) (4=1) (5=1) (ELSE=SYSMIS) INTO amount_meatd1.

EXECUTE.

RECODE amount_meatd (0=1) (1=1) (2=1) (3=2) (4=2) (5=2) (ELSE=SYSMIS) INTO amount_meatd2.

EXECUTE.

**recode frequency dairy dessert (dairy_dessert).*

RECODE dairy_dessert (1=1) (2=1) (3=1) (4=2) (5=3) (6=4) (7=5) (8=6) (9=7) (10=8) (MISSING=SYSMIS) INTO freq_dairy_dessert.

**recode frequency egg intake (freq_eggd).*

RECODE freq_eggd (1=1) (2=1) (3=1) (4=2) (5=1) (6 thru highest=1) (MISSING=SYSMIS) INTO freq_eggd1.

EXECUTE.

RECODE freq_eggd (1=1) (2=1) (3=1) (4=1) (5=2) (6 thru highest=1) (MISSING=SYSMIS) INTO freq_eggd2.

EXECUTE.

RECODE freq_eggd (1=1) (2=1) (3=1) (4=1) (5=1) (6 thru highest=2) (MISSING=SYSMIS) INTO freq_eggd3.

EXECUTE.

recode frequency pasta (freq_pastad).

RECODE freq_pastad (1=1) (2=1) (3=2) (4=1) (5 thru highest=1) (MISSING=SYSMIS) INTO freq_pastad1.

EXECUTE.

RECODE freq_pastad (1=1) (2=1) (3=1) (4=2) (5 thru highest=1) (MISSING=SYSMIS) INTO freq_pastad2.

EXECUTE.

RECODE freq_pastad (1=1) (2=1) (3=1) (4=1) (5 thru highest=2) (MISSING=SYSMIS) INTO freq_pastad3.

EXECUTE.

recode frequency fish (freq_fishd).

RECODE freq_fishd (1=1) (2=1) (3=2) (4=1) (5 thru highest=1) (MISSING=SYSMIS) INTO freq_fishd1.

EXECUTE.

RECODE freq_fishd (1=1) (2=1) (3=1) (4=2) (5 thru highest=1) (MISSING=SYSMIS) INTO freq_fishd2.

```
EXECUTE.

RECODE freq_fishd (1=1) (2=1) (3=1) (4=1) (5 thru highest=2) (MISSING=SYSMIS) INTO
    freq_fishd3.

EXECUTE.

* recode frequency peanuts (freq_peanutsd).

RECODE freq_peanutsd (1=1) (2=2) (3=2) (4 thru highest =1) (MISSING=SYSMIS) INTO
    freq_peanutsd1.

RECODE freq_peanutsd (1=1) (2=1) (3=1) (4 thru highest =2) (MISSING=SYSMIS) INTO
    freq_peanutsd2.

*recode frequency cheese on bread (cheese_on_bread).

RECODE cheese_on_bread (1=1) (2=1) (3=1) (4=2) (5=3) (6=4) (7=5) (8=6) (9=7) (10=8)
    (MISSING=SYSMIS) INTO  freq_cheese_on_bread.

EXECUTE.

*recode amount bread with cheese (bread_with_cheese).

RECODE bread_with_cheese (1=1) (2=1) (3=2) (4 thru highest = 3) (MISSING=SYSMIS)
    INTO amount_bread_with_cheese.

EXECUTE.

RECODE bread_with_cheese (1=1) (2=1) (3=2) (4 thru highest = 1) (MISSING=SYSMIS)
    INTO amount_bread_with_cheesed1.

EXECUTE.

RECODE bread_with_cheese (1=1) (2=1) (3=1) (4 thru highest = 2) (MISSING=SYSMIS)
    INTO amount_bread_with_cheesed2.

EXECUTE.
```

3. **Step: Calculate the predicted probabilities by the validated regression equation with shrinkage factor of 0.92. by Wijnhoven et al. 2018**

*Pay attention to the minus sign before each regression coefficient.

COMPUTE z2 = 0.92*19.361 +0.106*0.92*weight_adj -0.326*0.92*amount_slice_breadd1 - 1.175*0.92*amount_slice_breadd2 -2.750*0.92*amount_slice_breadd3 - 0.344*0.92*amount_milkd1 -1.681*0.92*amount_milkd2 -1.326*0.92*amount_meatd1 - 3.074*0.92*amount_meatd2 -0.175*0.92*freq_dairy_dessert -0.256*0.92*freq_eggd1 - 0.636*0.92*freq_eggd2 -1.480*0.92*freq_eggd3 -0.432*0.92*freq_pastad1 - 0.713*0.92*freq_pastad2 -1.409*0.92*freq_pastad3 -0.454*0.92*freq_fishd1 - 0.757*0.92*freq_fishd2 -1.100*0.92*freq_fishd3 -0.393*0.92*freq_peanutsd1 - 0.888*0.92*freq_peanutsd2 -0.177*0.92*freq_cheese_on_bread -0.654*0.92*amount_bread_with_cheesed1 -1.214*0.92*amount_bread_with_cheesed2.

EXECUTE.

COMPUTE predprob = 1/(1 + EXP(-z2)) .

EXECUTE.

VARIABLE LABELS predprob 'predicted probability protein intake < 1.0 g/kg BW/d'.

*Verify results by comparing with the results of the online tool (https://proteinscreener.nl/#/)

References:

Berner LA, Becker G, Wise M, Doi J. Characterization of dietary protein among older adults in the United States: amount, animal sources, and meal patterns. Journal of the Academy of Nutrition and Dietetics. 2013;113(6):809-15.

Wijnhoven HAH, Elstgeest LEM, de Vet HCW, Nicolaou M, Snijder MB, Visser M. Development and validation of a short food questionnaire to screen for low protein intake in community-dwelling older adults: The Protein Screener 55+ (Pro55+). PLoS One. 2018;13(5):e0196406.

Supplementary Material 2: Intake of energy and macronutrients for total sample and stratified by sex based on the 3-day dietary record

Dietary Intake	Men (n=55)	Women (n=89)	p-value
**Energy [kcal/d]**	1976.7±504.1	1642.3±340.0	<0.001
**Carbohydrates [E%]**	40.8 ±7.7	42.5 ±6.8	0.126
**Fat [E%]**	37.7 ±6.8	39.4 ±6.7	0.223
**Protein [g/d]**	73.9 ±17.1	63.8 ± 16.3	0.001
**Protein [E%]**	16.0 ±2.8	15.6 ±3.1	0.264
**Protein [g/kg aBW/d]**	1.0 ±0.2	1.0 ±0.3	0.140
**Protein <1.0 g/kg aBW/d [%]**	40.0	39.3	0.936
**Protein <0.8 g/kg aBW/d [%]**	16.4	18.0	0.804

Continuous variables: mean ± standard deviation, Mann-Whitney-U-Test; categorical/nominal variables: Chi²-Test; aBW adjusted bodyweight

Supplementary Material 3: Responses to the Protein Screener questions [%] for the German and the original Dutch (Longitudinal Aging Study Amsterdam (LASA)) validation study

	German Study (n=144)	LASA (n=1348)
**Bread**		
< 3 slices	52.8	33.6
3 slices	20.8	25.3
4 slices	18.8	24.1
≥ 5 slices	7.6	17.1
**Milk**		
< 1 glass	47.9	35.5
1 glass	21.5	43.3
≥ 2 glasses	30.6	21.4
**Meat**		
Small portion	41.7	16.2
Medium portion	31.3	51.1
Big portion	27.1	32.7
**Dairy Products**		
<1 day/week	27.8	28.8
1 day/week	8.3	4.7
2 days/week	9.0	6.2
3 days/week	9.7	7.6
4 days/week	2.1	5.9
5 days/week	7.6	10.0
6 days/week	4.9	11.3
7 days/week	30.6	25.7

**Eggs**		
<1 day/week	31.9	28.3
1 day/week	15.3	28.5
2 days/week	25.0	21.6
≥3 days/week	27.8	21.7
**Pasta**		
≤1 day/4 weeks	11.1	22.0
2-3 days/4 weeks	21.5	26.6
1 day/week	17.4	32.4
≥1 day/week	50.0	19.0
**Fish**		
≤1 day/4 weeks	13.9	25.4
2-3 days/4 weeks	19.4	23.0
1 day/week	34.0	31.6
≥2 days/week	32.6	20.0
**Nuts**		
<1 day/4 weeks	18.1	25.3
1-3 days/4 weeks	20.8	28.4
≥1 day/week	61.1	46.3
**Bread with Cheese**		
<1 day/week	13.9	11.5
1 day/week	4.9	3.5
2 days/week	13.9	8.3
3 days/week	14.6	9.1
4 days/week	10.4	9.4
5 days/week	9.7	13.7
6 days/week	15.3	8.8

7 days/week	17.4	35.6
**Bread with Cheese**		
≤1 Slice	38.9	46.8
2 Slices	38.2	38.7
≥3 Slices	22.9	14.5
